# Effects of different thermal processing methods on amino acid, fatty acid, and volatile flavor substance contents of Aohan millet (*Golden seedling millet*)

**DOI:** 10.1002/fsn3.4409

**Published:** 2024-09-17

**Authors:** Likun Cheng, Shuang Qu, Yueying Yun, Yan Ren, Fucheng Guo, Yakun Zhang, Guoze Wang

**Affiliations:** ^1^ College of Life Science and Technology Inner Mongolia University of Science and Technology Baotou Inner Mongolia P.R. China

**Keywords:** amino acids, Aohan millet, fatty acids, thermal processing method, volatile flavor compounds

## Abstract

Aohan millet has been cultivated for 8000 years and has rich nutritional value, such as high‐quality fatty acids and amino acids. Thermal processing is a conventional approach to food preparation. However, the effect of thermal processing on the formation of flavor substances in millet has not been clarified. Therefore, in this study, the effects of three different thermal processing techniques, namely, steaming, stir‐frying, and puffing, on the amino acids, fatty acids, and volatile flavor substances of Aohan millet were investigated using high‐speed automatic amino acid analyzer, headspace solid‐phase microextraction method, combined with gas chromatography–mass spectrometry (HS‐SPME/GC–MS) and gas chromatography (GC) with Aohan millet from Inner Mongolia as the raw material. All three thermal processing methods notably reduced the levels of protein, starch, polyphenols, and flavonoids in Aohan millet when compared to the raw millet (*p* < .05). Amino acid and fatty acid contents demonstrated an increase in fried and puffed millet relative to steamed millet, with notable distinctions in amino acid and fatty acid contents between these two groups. Following the steaming process, there was a significant increase in the flavor compounds of Aohan millet, rising from 53 to 80, while this increase was not observed in the other two groups. The correlation analysis suggested that the formation of flavor compounds was predominantly influenced by the types and levels of amino acids. The study suggested that different heat treatments affected the amino acid, fatty acid, and more significantly flavor material content and composition of Aohan millet. In conclusion, steaming treatment could retain more nutrients and richer flavor substances; while puffing treatment would enhance amino acid and fatty acid content, which provides a fundamental basis for scientifically guided processing and rational culinary application of Aohan millet.

## Graphical abstract

Puffed millet shows reduced fat and starch levels, rendering it a suitable choice for individuals seeking weight loss. Fried and puffed millet exhibited increased amino acid and fatty acid content compared to steamed millet. The correlation analysis suggested that the formation of flavor compounds was predominantly influenced by the types and levels of amino acids.

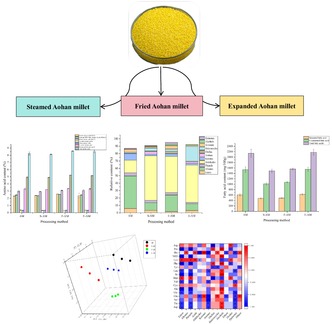

## 1 INTRODUCTION

Aohan Millet, cultivated in Aohan Banner, Chifeng City, Inner Mongolia Autonomous Region, is renowned as the “birthplace of world Xiaomi”. Historical records indicate that millet has been cultivated in this region for over 8000 years, making it one of the earliest areas for millet cultivation and consumption globally (Jia et al., 2016) The region offers ample sunshine, significant day–night temperature fluctuations, moderate precipitation, and topographic features such as hills, slopes, and sandy soil, which collectively provide a unique environment conducive to grain growth (Wang, Wang, et al., 2021). Aohan millet is grown organically, utilizing organic fertilizers sourced from cattle, sheep, and other livestock, alongside environmentally friendly pest control methods. Furthermore, the soil in Aohan Banner is rich in well‐balanced minerals like iron and phosphorus, contributing to the inherent high‐quality attributes of Aohan millet (Feng, 2020). The Aohan Dryland Farming System has earned recognition as a ‘Globally Important Heritage Systems’ site by the Food and Agriculture Organization (FAO) (Min & Zhang, 2019). In the local Chinese market, Aohan millet has seen a surge in sales as a premium product and is known for its beneficial effects on blood sugar and lipid levels, establishing its reputation as a cereal with both medicinal and dietary values (Jones, 2017; Sabuz et al., 2023; Vedamanickam et al., 2020).

Thermal processing is a conventional approach to food preparation. This process involves heating food, leading to the denaturation of proteins, gelatinization of starch, and the disruption of cellular tissue structure. These changes result in the release of cellular contents, rendering the food more easily digestible and enhancing its nutritional value (Zhan et al., 2018). Thermal processing also offers various advantages, including pathogen inactivation, reduction of natural toxins, extension of shelf life, improved palatability, taste, texture, and flavor, as well as enhanced functional characteristics (van Boekel et al., 2010). Common thermal processing techniques for cereals include steaming, boiling, stir‐frying, roasting, and puffing. These methods can alter the color, texture, and flavor of food, making it more appealing. Moreover, thermal processing can modify the nutritional composition of food, including fat content, vitamins, and minerals (Jongyingcharoen & Ahmad, 2014). Different thermal processing methods exert varying effects on the nutritional properties and functional composition of food. For instance, cooking can dissolve water‐soluble substances, such as soluble sugars (Yang et al., 2019), water‐soluble vitamins (Bureau et al., 2015), and trace elements (Koplík et al., 2004), in food, leading to a significant reduction in their content. Conversely, steaming does not bring the grains into direct contact with water, resulting in fewer effects on the water‐soluble components of the food. Research by Pradeep indicates that both atmospheric pressure steaming and high‐pressure steaming can enhance the content of total phenols, total flavonoids, tannins, and ferulic acid in millet, with high‐pressure steaming having a more pronounced effect (Pradeep & Guha, 2011). The preparation of expanded grains relies on high‐temperature, short‐time treatment (HTST), which causes the endosperm to expand due to the generation of steam within it (Dharmaraj et al., 2012). Huang observed that expansion treatment significantly enhances antioxidant capacity and minimizes the loss of proteins, amino acids, and other components compared to alternative thermal processing methods (Huang et al., 2023). Shabir compared nutrient composition and antioxidant differences in brown rice under three treatments (raw, boiled, and expanded), demonstrating that expansion treatment improves the nutritional value of brown rice, while boiled treatment increases mineral content (Mir et al., 2016).

Currently, both domestic and international research on millet processing primarily focus on methods, such as steaming and boiling, with limited exploration of frying, puffing, and other methods. This limitation was due to the challenge of processing millet, characterized by its small particle size compared to other cereals. However, as living standards improve, the processing of millet has become more and more diversified, and it was important to understand the effect of thermal processing methods on the nutritional properties of food and the formation of flavor. In addition, flavor substance research was focused on determining the relationship between key volatile compounds and their sensory properties. The flavor of millet was developed mainly during various processing treatments, especially heat treatment processing, as well as precursor flavor substances and enzymes from natural millet that contribute to flavor formation. The flavor of processed millet was a complex combination of volatile aromas associated with heat. These were the innovative points of the present study and underscoring the significance of understanding the impact of thermal processing methods on the nutritional properties of food. Consequently, this study employs steaming, frying, and puffing as three thermal processing methods for Aohan millet. The research investigates the impact of these methods on nutrient content, functional composition, and related factors, offering theoretical guidance for the scientific processing and rational culinary utilization of Aohan millet, thereby enhancing its application potential.

## 2 MATERIALS AND METHODS

### 2.1 Chemicals and materials

Aohan Millet was commercially sourced. α‐Amylase, protease, and amyloglucosidase were procured from Aladdin Biochemical Technology Co., Ltd. (Shanghai, China). Folin Reagent was obtained from Solarbio Science and Technology Co., Ltd (Beijing, China). Gallic acid was acquired from Kramar Reagent Co., Ltd (Shanghai, China). Rutin served as the standard. A set of 37 fatty acid mixture standards was purchased from the Sigma Company. All other reagents used were of analytical purity.

### 2.2 Instruments and equipment

The following equipment and instruments were employed: K9840 automatic Kjeldahl nitrogen analyzer (Haineng Future Technology Group Co., Ltd), SXT‐06 Soxhlet extractor (Shanghai Hongji Instrument Equipment Co., Ltd), L‐8900 amino acid analyzer, 6890 N‐G5795B Gas Chromatograph Mass Spectrometer (Agilent), Ultraviolet spectrophotometer, XLR high‐speed refrigerated centrifuge (Thermo Fisher Technology (China) Co., Ltd).

### 2.3 Methods

#### 2.3.1 Heat processing of Aohan millet

Millet grains with no presence of moths, debris, mildew, and exhibiting full freshness were selected as raw materials. These grains underwent thorough cleaning, involving washing 2 to 3 times, before further processing. The processing of Aohan millet included three methods:

- Steamed Aohan millet, S‐AM: The prepared Aohan millet was steamed under boiling water (95°C) for approximately 30 min, adhering to a millet‐to‐water ratio of 1:1 (mass ratio) (Bai et al., 2022).
- Fried Aohan millet, F‐AM: Processed Aohan millet was dried at 37°C and then fried in a hot pan (300 W) for 30 min without the addition of oil (Bi et al., 2021).
- Expanded Aohan millet, E‐AM: Processed Aohan millet was dried at 37°C and subjected to puffing using a popcorn machine.

After the heating process, the millet was allowed to cool to room temperature, following which it was ground using a grinder and sifted through a 200‐mesh sieve. The millet flour was then individually packed in sealed bags and stored at −40°C for future use.

#### 2.3.2 Nutrient determination

##### Determination of polyphenol content

The polyphenol content of the extract was assessed using the Folin–Ciocalteu method, as described by Adom et al. (2003). In brief, 5 mL of sample extract (1 mg/mL) was combined with 1 mL of Folin–Ciocalteu reagent after 5 min of incubation. This mixture was followed by the addition of 2 mL of 20% sodium carbonate (Na_2_CO_3_), and the final volume was adjusted to 10 mL with distilled water. The resulting mixture stood in darkness for an additional 60 min, after which absorbance was measured at 765 nm. The polyphenol content was determined from a calibration curve and expressed as milligrams of gallic acid equivalent per gram (mg GAE/g) of dry weight. The linear regression equation was *y* = 10.597*x* + 0.0266, with *R*_2_ = 0.9992.

##### Determination of flavonoid content

The flavonoid content of the sample extract was assessed via the aluminum chloride (AlCl_3_) colorimetric method, based on Adom's protocol (Adom et al., 2003), with slight adjustments. Specifically, 5 mL of the crude extract was mixed with 0.7 mL of 5% sodium nitrite (NaNO_2_) solution. After 5 min of incubation, 0.7 mL of 10% AlCl_3_ solution was added, followed by 5 mL of 1 mol/L sodium hydroxide (NaOH) solution. The final volume of the mixture was adjusted to 25 mL with 60% ethanol. After 15 min of standing, absorbance was measured at 510 nm. The total flavonoid content was calculated from a calibration curve and expressed as milligrams of rutin equivalent per gram (mg RE/g) of dry weight. The linear regression equation was *y* = 9.0825*x* + 0.017, with *R*_2_ = 0.999.

#### 2.3.3 Determination of amino acid content

Amino acid contents were determined following Huang's method (Huang et al., 2019) with minor modifications. Approximately 300 mg of Aohan millet powder was weighed and placed in a digestion tube. Subsequently, 10 mL of 6 mol/L hydrochloric acid (HCl) solution was added, followed by 2 min of ultrasonication. The tube was sealed under a nitrogen atmosphere. After cooling, the digestion tube was shaken at room temperature, and its cap was opened. The solution was then diluted with ultrapure water in a 100 mL volumetric flask and thoroughly mixed. It was filtered through a 0.45‐μm inorganic filter membrane, and 2.5 mL of this filtrate was transferred into a 25 mL volumetric flask and further diluted with ultrapure water. This solution was again filtered using a 0.45‐μm inorganic filter membrane. The resulting filtrate was used for detection and analysis on a LA8080 high‐speed automatic amino acid analyzer.

#### 2.3.4 Determination of fatty acid content

Fatty acid content determination was performed according to Honicky's method (Honicky et al., 2020), with minor modifications. After extracting the fat from the sample, fat saponification and fatty acid methylation were carried out. In the fat extract, 2 mL of 2% sodium hydroxide in methanol solution was added and subjected to a water bath at 85°C for 30 min. Subsequently, 3 mL of 14% boron trifluoride (BF_3_) in methanol solution was added to the water bath at 85°C for an additional 30 min. Following the water bath, and once the temperature had cooled to room temperature, 1 mL of n‐hexane was introduced into the centrifuge tube. After shaking for 2 min, the mixture was left undisturbed for an hour to facilitate stratification. A volume of 100 μL of the supernatant was taken and adjusted with n‐hexane to a final volume of 1 mL. This solution was then filtered through a 0.45‐μm filter membrane and analyzed using the appropriate machine. The gas chromatography conditions included a TG‐FAME column measuring 50 m × 0.25 mm × 0.20 μm, with a heating procedure that was initiated at 80°C for 1 min, followed by a heating rate of 20°C/min to 160°C, where it was held for 1.5 min, and ultimately a heating rate of 3°C/min to 250°C, which was maintained for 3 min. The inlet temperature was set at 270°C, utilizing nitrogen as the carrier gas with a flow rate of 0.63 mL/min. The injection was shunt‐based with a 100:1 shunt ratio, and a hydrogen flame ionization detector (FID) was employed with a detector temperature of 270°C.

#### 2.3.5 Determination of volatile flavor compounds

Volatile flavor compounds were determined following methods described by Yaqub and Gay (Gay et al., 2020; Yaqub et al., 2020). Initially, saturated sodium chloride (NaCl) was added to a 20 mL headspace bottle and heated at 80°C for 30 min. After this period, a headspace microextraction injection needle was inserted into the headspace bottle, and the heating continued for another 30 min. Subsequently, the analysis was conducted with an inlet temperature of 250°C for 5 min. The chromatographic conditions were configured as follows: the column used was HP‐5MS, with dimensions of 30 m × 0.25 mm × 0.25 μm. The column temperature was initiated at 50°C for 2 min, then increased at a rate of 5°C/min to 180°C for 5 min, and finally, it was elevated at a rate of 10°C/min to 250°C for another 5 min. The inlet temperature was maintained at 250°C, and the transmission line temperature was 280°C. The carrier gas flow rate was 1.0 mL/min, and no shunt was utilized. For mass spectrometry, the ion source temperature was set to 230°C, the quaternary rod temperature to 150°C, and the mass spectrum operated in the electron ionization (EI) source mode with a full sweep range of 40–600.

#### 2.3.6 Statistics analysis

Data analysis was conducted using SPSS software (SPSS PASW 18.0). Duncan's multiple range test was used to separate means (*p* < .05). All presented data were given as the mean with the standard deviation. All tests were conducted in triplicate. Principal component analysis (PCA) was executed using Origin 2018 statistical software (Origin Lab, Northampton, MA, USA). Correlation analyses were performed using Spearman's rank correlation coefficient, which was a nonparametric statistical method suitable for non‐normally distributed data.

## 3 RESULTS AND DISCUSSION

### 3.1 Nutritional analysis of Aohan millet

Table 1 provides an overview of the basic nutrient composition of Aohan millet after undergoing three distinct thermal processing methods. Notably, all three processing methods led to a significant reduction (*p* < .05) in the protein content of Aohan millet when compared to the raw AM. However, there was no significant difference in protein content between steamed S‐AM and F‐AM (*p* > .05). Conversely, after steaming, the fat content of Aohan millet experienced a notable increase (*p* < .05), which can be attributed to the rupture of cell walls during heating, facilitating fat dissolution and augmenting its content. In contrast, both frying and puffing treatments resulted in a substantial reduction in fat content (*p* < .05). This reduction may be due to the breakdown and structural degradation of fat during frying and puffing, causing the release of fat. Furthermore, starch content displayed a significant variation (*p* < .05) among these three processing methods, with reductions of 18.32%, 10.18%, and 59.82% in S‐AM, F‐AM, and E‐AM, respectively, when compared to AM. There was aqueous leaching of amylose or degradation of the starch granules leading to consequent reduction in starch content, with the highest starch loss observed in the expansion treatment, given its pressurized nature (Saha & Roy, 2020).

**TABLE 1:** Effects of different thermal processing methods on basic nutrients of Aohan millet.

Processing method	Protein content (g/100 g)	Fat content (g/100 g)	Starch content (g/100 g)
AM	11.56 ± 0.30^a^	3.16 ± 0.06^b^	65.61 ± 0.73^a^
S‐AM	7.48 ± 0.30^b^	5.45 ± 0.39^a^	53.59 ± 0.45^c^
F‐AM	7.45 ± 0.25^b^	0.21 ± 0.08^d^	58.93 ± 0.64^b^
E‐AM	4.39 ± 0.30^c^	1.64 ± 0.32^c^	26.36 ± 1.73^d^

*Note*: Mean ± SD (*n* = 3). Means with different superscripts (a, b) in the same row are significantly different (*p* < .05).

Abbreviations: AM, Raw Aohan millet; E‐AM, Expanded Aohan millet; F‐AM, Fried Aohan millet; S‐AM, Steamed Aohan millet.

### 3.2 Analysis of the content of functional components of Aohan millet

Functional components, such as polyphenols and flavonoids, are known for their anti‐inflammatory and antiaging properties (Bai et al., 2021; Wang, Cao, et al., 2021). Table 2 illustrates the impact of three thermal processing methods on the content of functional components in Aohan millet. It was evident that thermal processing led to a significant reduction (*p* < .05) in both polyphenol and flavonoid contents of Aohan millet. The polyphenol content of F‐AM exhibited the least decrease, while the flavonoid content of expanded E‐AM experienced the least reduction. Conversely, S‐AM demonstrated the most substantial decrease in both polyphenols and flavonoids, with a reduction of 56.64% and 45.06%, respectively. This finding aligns with the results of a study by Wu, which revealed that four heating methods (boiling, roasting, extruding, and pressure cooking) led to decreased polyphenol, flavonoid, and saponin content in quinoa, consistent with the outcomes of this experiment (Wu et al., 2021). In a study by Das, it was determined that the optimal extraction conditions for polyphenols in tea were 80°C for 20 min, with polyphenol content decreasing as temperature and time increased. The results indicate that all three heat processing methods (steaming, frying, and puffing) resulted in polyphenol loss in millet due to the higher temperatures involved (Das & Eun, 2018). Shigihalli also checked the popping effect on the nutritional and antinutritional profiles of finger millet. The result showed a decrease in fat content and the antinutrients’ content like trypsin inhibitor activity, tannins, and phytic acid (Shigihalli et al., 2018). Notably, the highest polyphenol content was observed in E‐AM due to the rapid preparation of puffed millet, involving a shorter duration of heat exposure and creating a porous structure that aids in the release of polyphenolic substances.

**TABLE 2:** Effects of different thermal processing methods on functional component content of Aohan millet.

Processing method	Polyphenol content (mg/100 g)	Flavonoid content (mg/100 g)
AM	344.04 ± 3.29^a^	58.85 ± 2.15^a^
S‐AM	149.16 ± 7.17^d^	32.33 ± 2.46^c^
F‐AM	222.96 ± 4.71^c^	49.66 ± 1.60^b^
E‐AM	303.24 ± 28.72^b^	30.03 ± 2.80^d^

*Note*: Mean ± SD (*n* = 3). Means with different superscripts (a, b) in the same row are significantly different (*p* < .05).

Abbreviations: AM, Raw Aohan millet; E‐AM, Expanded Aohan millet; F‐AM, Fried Aohan millet; S‐AM, Steamed Aohan millet.

### 3.3 Effects of heating–processing on amino acids of Aohan millet

Amino acids play a crucial role in enhancing the flavor and color of food, making them a significant determinant of food quality (Yao et al., 2006). Table 3 outlines the impact of different thermal processing methods on the amino acid composition and content of Aohan millet. It was evident that Aohan millet contains 18 amino acids, with a total content ranging from 8.13% to 8.60%. Glutamic acid was the most abundant amino acid, followed by leucine, proline, and aspartic acid. The essential amino acid and total amino acid contents of fried and puffed millet were elevated compared to raw millet, although the changes were not statistically significant (*p* > .05). Studies have indicated that increased amino acid content can provide several benefits, including relaxation and nervous system benefits (Nathan et al., 2006). Millet contains eight essential amino acids, with leucine, phenylalanine, and isoleucine being the top three in terms of content. Histidine was an essential amino acid for infants and young children, and its content remains largely unaffected by thermal processing. Glutamic acid holds the highest content among nonessential amino acids, accounting for 1.81% to 1.96%. It was worth noting that the proportion of essential amino acids to the total mass of amino acids in each group ranged from 39.05% to 40.19%, while the proportion of essential amino acids to the total mass of nonessential amino acids ranged from 64.06% to 67.19%, closely aligning with the evaluation criteria of the FAO/WHO (World Health Organization) amino acid model (Herreman et al., 2020).

**TABLE 3:** Effects of different thermal processing methods on amino acid content of Aohan millet.

Amino acid types	Amino acid content (%)			
	AM	S‐AM	F‐AM	E‐AM
Sweet amino acids (SAA)				
Ala	0.78 ± 0.00^c^	0.78 ± 0.01^c^	0.85 ± 0.00^a^	0.82 ± 0.21^b^
Gly	0.20 ± 0.00^a^	0.19 ± 0.01^b^	0.21 ± 0.01^a^	0.10 ± 0.01^a^
Ser	0.41 ± 0.00^ab^	0.39 ± 0.00^b^	0.42 ± 0.00^a^	0.41 ± 0.01^ab^
Thr*	0.33 ± 0.00^b^	0.32 ± 0.00^c^	0.35 ± 0.00^a^	0.34 ± 0.01^b^
Pro	0.60 ± 0.01^c^	0.72 ± 0.01^b^	0.76 ± 0.00^a^	0.71 ± 0.07^a^
Total	2.32 ± 0.01^d^	2.40 ± 0.03^c^	2.59 ± 0.01^a^	2.38 ± 0.31^b^
Sour and MSG‐like amino acids (SMAA)				
Glu	1.89 ± 0.04^ab^	1.81 ± 0.01^b^	1.96 ± 0.01^a^	1.89 ± 0.06^ab^
Asp	0.57 ± 0.00^a^	0.55 ± 0.00^b^	0.59 ± 0.00^a^	0.58 ± 0.01^a^
Total	2.46 ± 0.04^ab^	2.36 ± 0.01^b^	2.55 ± 0.01^a^	2.47 ± 0.07^ab^
Bitter amino acids (BAA)				
Val*	0.42 ± 0.01^bc^	0.41 ± 0.01^c^	0.44 ± 0.00^a^	0.43 ± 0.01^ab^
Met*	0.20 ± 0.11^a^	0.16 ± 0.01^a^	0.10 ± 0.02^a^	0.17 ± 0.00^a^
Leu*	1.18 ± 0.02^b^	1.17 ± 0.00^b^	1.27 ± 0.00^a^	1.22 ± 0.04^ab^
Ile*	0.34 ± 0.01^a^	0.34 ± 0.00^a^	0.37 ± 0.01^a^	0.36 ± 0.01^a^
Phe*	0.50 ± 0.00^b^	0.51 ± 0.00^b^	0.55 ± 0.00^a^	0.52 ± 0.01^b^
His	0.17 ± 0.00^a^	0.16 ± 0.00^a^	0.17 ± 0.00^a^	0.17 ± 0.01^a^
Arg*	0.22 ± 0.00^a^	0.20 ± 0.01^b^	0.20 ± 0.01^b^	0.21 ± 0.01^ab^
Total	3.03 ± 0.15^a^	2.95 ± 0.03^a^	3.10 ± 0.04^a^	3.09 ± 0.10^a^
Tasteless amino acids				
Lys*	0.14 ± 0.00^a^	0.12 ± 0.01^b^	0.10 ± 0.00^c^	0.09 ± 0.00^c^
Tyr	0.22 ± 0.03^ab^	0.19 ± 0.03^b^	0.18 ± 0.01^b^	0.26 ± 0.01^a^
Total	0.36 ± 0.03^a^	0.31 ± 0.04^ab^	0.28 ± 0.01^b^	0.35 ± 0.01^a^
Other amino acids				
Cys	0.13 ± 0.01^a^	0.14 ± 0.01^a^	0.12 ± 0.01^a^	0.12 ± 0.01^a^
NH_3_	0.20 ± 0.01^b^	0.20 ± 0.00^ab^	0.22 ± 0.01^a^	0.21 ± 0.01^ab^
Total	0.33 ± 0.02^ab^	0.34 ± 0.01^a^	0.34 ± 0.02^ab^	0.33 ± 0.02^b^
Essential amino acids	3.32 ± 0.14^a^	3.22 ± 0.01^a^	3.37 ± 0.04^a^	3.33 ± 0.09^a^
Nonessential amino acid	4.94 ± 0.08^b^	4.91 ± 0.04^b^	5.24 ± 0.03^a^	5.19 ± 0.08^a^
Total amino acids	8.26 ± 0.23^ab^	8.13 ± 0.06^b^	8.60 ± 0.06^a^	8.52 ± 0.18^ab^
Essential Amino Acids/Total amino acids	40.19 ± 0.61^a^	39.61 ± 0.10^ab^	39.10 ± 0.12^b^	39.05 ± 0.27^b^
Essential Amino Acids/Nonessential amino acid	67.19 ± 1.71^a^	65.58 ± 0.28^ab^	64.22 ± 0.33^b^	64.06 ± 0.72^b^

*Note*: Mean ± SD (*n* = 3). Means with different superscripts (a, b) in the same row are significantly different (*p* < .05).

Abbreviations: AM, Raw Aohan millet; E‐AM, Expanded Aohan millet; F‐AM, Fried Aohan millet; S‐AM, Steamed Aohan millet.

* Indicates essential amino acids.

**FIGURE 1:**
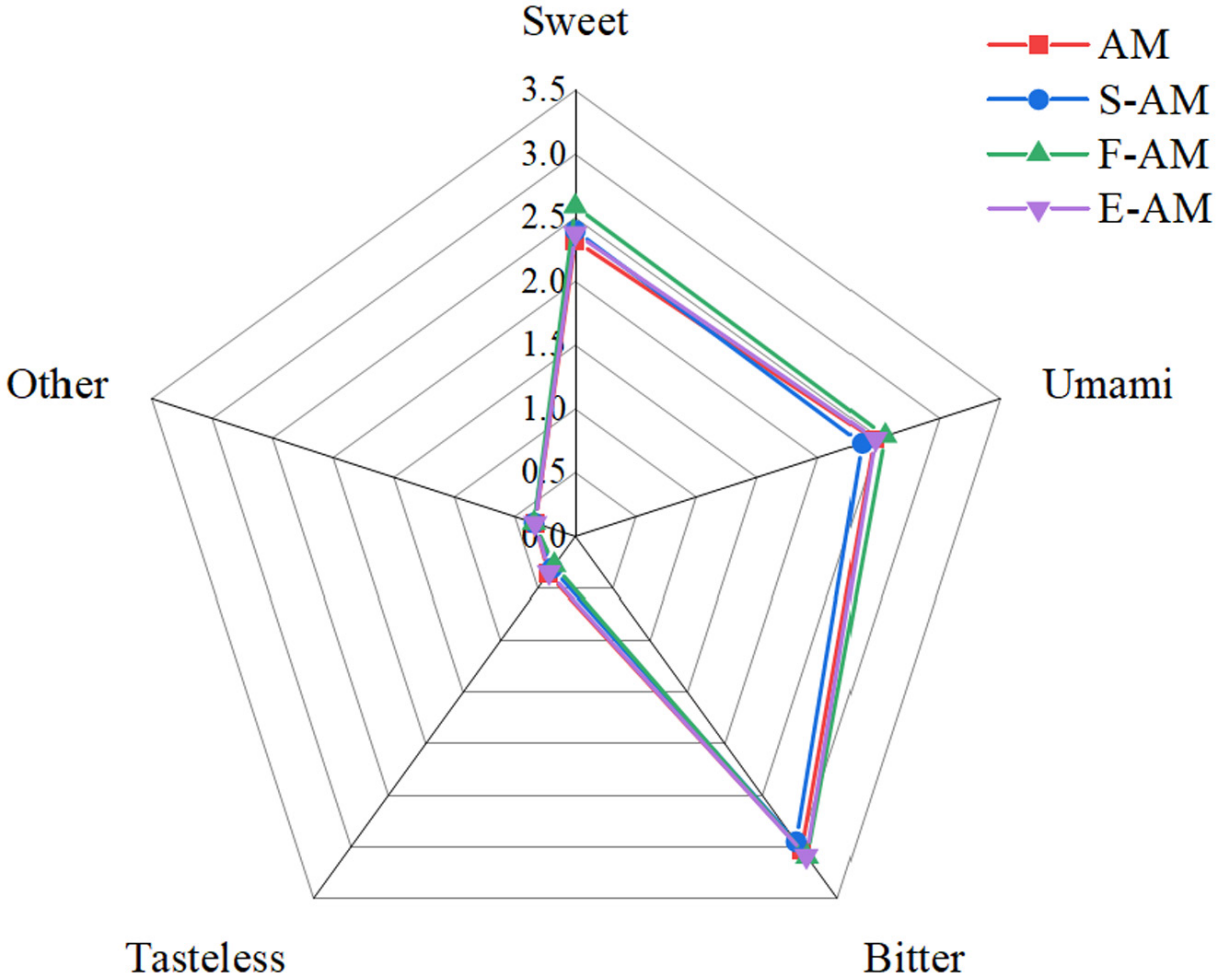
Radar graph of amino acids in Aohan millet under different hot processing methods. AM, Aohan millet; F‐AM, Fried Aohan millet; E‐AM, Expanded Aohan millet; S‐AM, Steamed Aohan millet. Sweet = Ala+Gly + Ser + Thr + Pro; Umami = GLU + ASP; Bitter = Val + Met+Leu + Ile + Phe + His+Arg; Tasteless = Lys + Tyr; Other = Cys + NH_3_.

Amino acids, such as alanine, glycine, serine, and threonine, impart a sweet taste, while glutamic and aspartic acids contribute to a pleasant fresh taste. Valine, methionine, leucine, isoleucine, phenylalanine, histidine, and arginine are associated with a bitter taste (Li‐Chan & Cheung, 2010). As depicted in Table 3, the content of sweet amino acids was significantly higher (*p* < .05) in F‐AM, along with elevated levels of fresh and bitter amino acids. Conversely, S‐AM exhibited the least amount of bitter amino acids, suggesting that the steaming treatment reduced the bitter flavor. Figure 1 further demonstrates that F‐AM contained a higher proportion of sweet, fresh, and bitter amino acids, while raw millet contained more odorless amino acids. Consequently, Aohan millet exhibits improved flavor characteristics to some extent after undergoing thermal processing, with the most noticeable enhancement observed in fried millet.

### 3.4 Gas‐phase analysis of the fatty acid composition and content of Aohan millet

Gas‐phase analysis of the fatty acid composition and content of Aohan millet revealed essential findings, as elucidated in Table 4. It was evident that saturated fatty acids are 28.26% of the total mass of total fatty acids, with palmitic acid being the highest (388.65 mg/100 g). Furthermore, unsaturated fatty acids contribute to 71.74% of the total fatty acid content, with linoleic acid being the most prevalent at 1208.70 mg/100 g, followed by oleic acid (258.35 mg/100 g). The millet is rich in fatty acids. Palmitic acid, stearic acid, oleic acid, linoleic acid, and linolenic acid are the main components of foxtail millet (Li et al., 2020), and it bears resemblance to this result. These findings underscore the high‐quality fat content in millet, particularly due to the presence of linoleic and oleic acids, which confer hypoglycemic and hypolipidemic properties, thereby promoting human health and averting diseases (Annor et al., 2015; Ren et al., 2021). It was noteworthy that both steaming and frying processes resulted in a significant (*p* < .05) decrease in the levels of saturated and unsaturated fatty acids relative to raw millet, while puffed millet exhibited a nonsignificant (*p* > .05) increase in both types of fatty acids. This phenomenon was attributed to the release of fatty acids from millet fat cells following high‐temperature treatment and puffing, leading to an expansion in the oil yield area and enhanced fatty acid production (Das & Eun, 2018).

**TABLE 4:** Effects of different thermal processing on fatty acid content of Aohan millet.

Fatty acid type	Fatty acid content (mg/100 g)			
	AM	S‐AM	F‐AM	E‐AM
Saturated fatty acid				
Decanoic acid (C15:0)	3.90 ± 0.28^a^	4.00 ± 0.28^a^	4.15 ± 0.21^a^	4.15 ± 0.21^a^
Palmitic acid (C16:0)	388.65 ± 26.66^a^	358.95 ± 15.06^a^	356.85 ± 8.27^a^	404.05 ± 18.60^a^
Stearic acid (C18:0)	142.30 ± 9.62^a^	80.55 ± 3.32^b^	87.75 ± 2.05^b^	145.55 ± 7.28^a^
Eicosanoic acid (C20:0)	42.10 ± 3.11^a^	22.50 ± 1.13^b^	24.70 ± 0.99^b^	42.80 ± 2.12^a^
Heneicosanoic Acid (C21:0)	ND	ND	ND	1.65 ± 2.33^a^
Behenic acid (C22:0)	15.60 ± 1.13^a^	9.00 ± 0.42^b^	9.55 ± 0.21^b^	16.00 ± 0.85^a^
Tricosanoic acid (C23:0)	5.45 ± 0.35^a^	5.40 ± 0.28^a^	4.95 ± 0.07^a^	5.80 ± 0.42^a^
Lignoceric acid (C24:0)	7.60 ± 0.42^a^	ND	ND	8.15 ± 0.49^a^
Total saturated fatty acid	605.60 ± 41.58a	480.40 ± 20.51b	487.95 ± 11.81b	628.15 ± 32.31a
Unsaturated fatty acid				
Oleic acid (C18:1n9c)	258.35 ± 17.47^a^	162.15 ± 6.86^b^	17.44 ± 0.43^c^	260.55 ± 13.22^a^
cis‐11‐Eicosenoic acid (C20:1)	6.45 ± 0.49^a^	3.95 ± 0.21^b^	4.25 ± 0.07^b^	6.70 ± 0.28^a^
Erucic acid (C22:1n9)	5.15 ± 0.49^b^	4.00 ± 0.14^c^	5.80 ± 0.14^b^	9.85 ± 0.49^a^
Linoleic acid (C18:2n6c)	1208.70 ± 83.86^a^	800.30 ± 33.66^b^	845.30 ± 18.38^b^	1217.10 ± 57.98^a^
αLinolenic acid (C18:3n3)	58.55 ± 4.03^a^	44.00 ± 1.98^b^	45.40 ± 0.99^b^	58.70 ± 2.83^a^
Total unsaturated fatty acids	1537.20 ± 106.35^a^	1014.40 ± 42.85^b^	1075.10 ± 23.90^b^	1552.90 ± 74.81^a^
Total fatty acids	2142.80 ± 147.93^a^	1494.80 ± 63.36^b^	1563.05 ± 35.71^b^	2181.05 ± 107.13^a^
Saturated fatty acids/total fatty acids	28.26 ± 0.01^d^	32.14 ± 0.01^a^	31.21 ± 0.04^b^	28.80 ± 0.07^c^
Unsaturated fatty acids/total fatty acids	71.74 ± 0.01^a^	67.86 ± 0.01^d^	68.78 ± 0.04^c^	71.20 ± 0.07^b^

*Note*: Mean ± SD (*n* = 3). Means with different superscript (a, b) in the same row are significantly different (*p* < .05).

Abbreviations: AM, Raw Aohan millet; E‐AM, Expanded Aohan millet; F‐AM, Fried Aohan millet; S‐AM, Steamed Aohan millet.

### 3.5 HS‐SPME‐GC–MS analysis of volatile components of Aohan millet

An HS‐SPME‐GC–MS analysis was conducted to examine the volatile components of Aohan millet under various heat treatments. The analysis employed the headspace solid‐phase microextraction (HS‐SPME) method, combined with gas chromatography–mass spectrometry (GC–MS). The chromatographic data were cross‐referenced with the NIST14 (National Institute of Standards and Technology) spectral library and manually analyzed. The outcomes presented in Table 5 demonstrate alterations in volatile flavor substances in Aohan millet following different heat treatments. The results indicated that Aohan millet contains 53 volatile flavor compounds, including 6 esters, 3 aldehydes, 9 ketones, 3 acids, 17 alkanes, 1 nitrogen‐containing compound, 6 aromatic compounds, 5 heterocyclic compounds, 1 alcohol, and 2 oxides. Of these compounds, 80, 50, and 55 volatile compounds were detected after steaming, frying, and puffing, respectively. Notably, the variety of volatile flavor substances in Aohan millet significantly expanded following the steaming process, with a notable increase in esters, aldehydes, nitrogen‐containing compounds, and heterocyclic compounds compared to raw millet. In steamed millet, the highest content of veratraldehyde was 55.50%, which contributes to a sweet flavor and enhances the taste of steamed millet. Meanwhile, fried and puffed millet exhibited the highest piperonal content at 34.76% and 39.14%, respectively, among the volatile compounds. A comparison with raw millet unveiled significantly higher levels of aldehydes, amines, olefins, alcohols, and heterocyclic compounds in all three heat processing methods, which can be attributed to the structural changes that occur due to heating and the consequent generation of certain compounds. The elevated aldehyde content may be on the one hand attributed to oxidative degradation of unsaturated fatty acids (Shi et al., 2019). However, it was worth noting that aldehydes play a role in the creation of the fragrant aroma of cooked millet (Bi et al., 2019). On the other hand, it may be explained by the occurrence of a Meladic reaction after heat treatment of millet, which promotes the production of aldehydes (Bai et al., 2022; Lykomitros et al., 2016). A previous study by Li. reported the identification of 62 volatile compounds in millet using HS‐SPME‐GC–MS, including 18 aldehydes, 6 alcohols, 9 ketones, 5 acids, 10 hydrocarbons, 10 benzene derivatives, and 4 other components (Li, Zhao, Liu, Zhang, et al., 2021). Similarly, Yang identified 8 fatty acids, 7 essential amino acids, and 59 volatile compounds in glutinous and non‐glutinous millet porridges, with components, such as 6 alcohols, 14 aldehydes, 22 alkanes, 4 ketones, 1 benzene, 8 acids and esters, 2 amines, 1 heterocyclic group, and 1 olefin contributing to the flavor profile (Yang et al., 2021). These findings align with the results of the current study.

**TABLE 5:** Effects of different thermal processing on volatile organic compounds of Aohan millet.

Compound number	Retention time (min)	Compound	CAS	Molecular formula	Relative content (%)			
					AM	S‐AM	F‐AM	E‐AM
Esters (20)								
VOC1	3.383	Acetic acid, dimethoxy‐, methyl ester	89–91‐8	C_5_H_10_O_4_	/	/	0.64	/
VOC2	4.651	2‐Propenoic acid, 3‐[4‐(acetyloxy)‐3‐methoxyphenyl]‐, methyl ester	2309‐08‐2	C_13_H_14_O_5_	/	/	0.16	/
VOC3	4.670	3,4‐Dimethyl‐2‐(3‐methyl‐butyryl)‐benzoic acid, methyl ester	71940–29‐9	C_15_H_20_O_3_	/	/	0.35	/
VOC4	4.684	1,4‐Benzenedicarboxylic acid, 2‐amino‐, dimethyl ester	5372‐81‐6	C_10_H_11_NO_4_	/	/	/	0.30
VOC5	4.688	1,2‐Benzenedicarboxylic acid, 4‐nitro‐, dimethyl ester	610–22‐0	C_10_H_9_NO_6_	0.20	/	/	0.18
VOC6	4.770	1,4‐Cyclopentadiene‐1‐carboxylic acid, 3‐[1‐(dimethylamino)ethylidene]‐, methyl ester	14485–75‐7	C_11_H_15_NO_2_	/	0.14	/	/
VOC7	4.792	1,2‐Benzenedicarboxylic acid, 4‐amino‐, dimethyl ester	51832–31‐6	C_10_H_11_NO_4_	/	0.24	/	/
VOC8	4.823	Benzoic acid, 2‐formyl‐4,6‐dimethoxy‐, 7‐formylhept‐2‐yl ester	312305–59‐2	C_18_H_24_O_6_	1.10	0.12	/	/
VOC9	6.660	Carbamic acid, methyl‐, 3‐methylphenyl ester	1129–41‐5	C_9_H_11_NO_2_	/	0.90	/	/
VOC10	6.730	Acetic acid, 4‐methylphenyl ester	140–39‐6	C_9_H_10_O_2_	/	0.27	/	/
VOC11	7.764	Methyl 2‐butynoate	23326–27‐4	C_5_H_6_O_2_	/	0.62	/	0.41
VOC12	8.454	di‐tert‐butyl dicarbonate	24424–99‐5	C_10_H_18_O_5_	/	/	/	0.80
VOC13	8.504	Propanoic acid, ethenyl ester	105–38‐4	C_5_H_8_O_2_	1.67	/	/	/
VOC14	14.805	t‐Butyl cinnamate	7042‐36‐6	C_13_H_16_O_2_	/	0.23	/	/
VOC15	18.888	2,2,4‐Trimethyl‐1,3‐pentanediol diisobutyrate	6846‐50‐0	C_16_H_30_O_4_	0.99	0.67	/	/
VOC16	19.452	Propanoic acid, 2‐methyl‐, 2‐methyl‐2‐propenyl ester	816–73‐9	C_8_H_14_O_2_	/	/	0.58	/
VOC17	21.527	Dimethyl phthalate	131–11‐3	C_10_H_10_O_4_	/	/	/	0.72
VOC18	24.886	Diethyl Phthalate	84–66‐2	C_12_H_14_O_4_	/	/	/	0.66
VOC19	25.453	3‐Methylbutyric acid, 2‐biphenyl‐4‐yl‐2‐oxoethyl ester	4376–32‐3	C_19_H_20_O_3_	1.08	/	/	/
VOC20	29.636	Phenyl salicylate	118–55‐8	C_13_H_10_O_3_	0.78	/	/	/
Total					5.82	3.19	1.73	3.07
Alkanes (32)								
VOC21	8.982	1,2‐Di(2,4,6‐trimethylphenyl)ethane	4674–23‐1	C_20_H_26_	/	0.20	/	/
VOC22	10.669	Cyclopentane, 1‐ethyl‐1‐methyl‐	16747–50‐5	C_8_H_16_	2.50	/	/	/
VOC23	11.008	Oxetane, 3‐(1‐methylethyl)‐	10317–17‐6	C_6_H_12_O	0.98	/	/	/
VOC24	11.035	Cyclopentane, propyl‐	2040‐96‐2	C8H1_6_	6.22	/	/	/
VOC25	11.989	Cyclopentane, methyl‐	96–37‐7	C_6_H_12_	/	/	2.14	0.51
VOC26	13.907	Cyclopentane, 1,1,3‐trimethyl‐	4516‐69‐2	C_8_H_16_	/	/	/	0.44
VOC27	14.706	Dodecane	112–40‐3	C_12_H_26_	3.64	1.26	2.61	0.74
VOC28	16.550	cis‐1‐Hydroxybicyclo[4.4.0]decane	3574‐58‐1	C_10_H_18_O	/	0.49	/	/
VOC29	17.458	Heptane, 2,2,4‐trimethyl‐	14720–74‐2	C_10_H_22_	/	0.24	/	/
VOC30	17.459	2,2,6,6‐Tetramethylheptane	40117–45‐1	C_11_H_24_	/	0.23	/	/
VOC31	17.475	Decane, 3‐methyl‐	13151–34‐3	C_11_H_24_	/	/	0.62	/
VOC32	17.475	Tridecane	629–50‐5	C_13_H_28_	0.88	/	/	0.51
VOC33	18.899	2,3’‐Bifuran, octahydro‐	73373–15‐6	C_8_H_14_O_2_	1.06	/	/	/
VOC34	19.324	Undecane, 5,5‐dimethyl‐	17312–73‐1	C_13_H_28_	/	/	/	0.18
VOC35	20.085	Decane, 2,5‐dimethyl‐	17312–50‐4	C_12_H_26_	/	/	/	1.37
VOC36	20.087	Tetradecane	629–59‐4	C_14_H_3_0	4.62	1.43	2.82	1.77
VOC37	22.576	Pentadecane	629–62‐9	C_15_H_32_	1.68	1.19	2.63	1.08
VOC38	22.960	Tetracyano‐p‐quinodimethane	1518‐16‐7	C_12_H_4_N_4_	/	0.39	/	/
VOC39	24.246	Hexadecane, 2‐methyl‐	1560–92‐5	C_17_H_36_	/	/	0.76	/
VOC40	24.253	Pentadecane, 3‐methyl‐	2882‐96‐4	C_16_H_34_	1.05	/	/	/
VOC41	24.923	Hexadecane	544–76‐3	C_16_H_34_	3.74	1.03	2.35	1.73
VOC42	26.041	Nonane, 2,2,4,4,6,8,8‐heptamethyl‐	4390–04‐9	C_16_H_34_	0.87	0.24	/	/
VOC43	26.044	Pentadecane, 2,6,10‐trimethyl‐	3892‐00‐0	C_18_H_38_	/	/	1.65	/
VOC44	26.181	Cyclohexane, tetradecyl‐	1795‐18‐2	C_20_H_40_	/	0.69	/	/
VOC45	27.161	Heptadecane	629–78‐7	C_17_H_36_	1.83	0.77	1.94	/
VOC46	27.175	Heptacosane	593–49‐7	C_27_H_56_	1.95	0.55	0.42	/
VOC47	27.176	Hexadecane, 7‐methyl‐	26730–20‐1	C_17_H_36_	/	/	1.06	/
VOC48	27.295	Pentadecane，2,6,10,14‐tetramethyl‐	1921‐70‐6	C_19_H_40_	4.30	0.51	/	0.79
VOC49	29.447	Octadecane	593–45‐3	C_18_H_38_	1.99	/	/	/
VOC50	29.630	Benzene, 1,1′‐[1,2‐ethanediylbis(oxy)]bis‐	104–66‐5	C_14_H_14_O_2_	4.65	1.19	2.14	/
VOC51	29.678	Pentadecane, 4‐methyl‐	2801‐87‐8	C_16_H_34_	1.91	/	/	/
VOC52	29.689	Tridecane, 2,5‐dimethyl‐	56292–66‐1	C_15_H_32_	/	/	1.07	/
Total					43.87	10.41	22.21	9.12
Phenols (6)								
VOC53	4.828	2‐Propenoic acid, 3‐(3,4‐dimethoxyphenyl)‐, (E)‐	14737–89‐4	C_11_H_12_O_4_	9.45	0.76	/	/
VOC54	17.870	Phenol, 5‐ethenyl‐2‐methoxy‐	621–58‐9	C_9_H_10_O_2_	/	3.26	1.27	4.97
VOC55	17.971	Phenol, 4‐(1‐methylpropyl)‐	99–71‐8	C_10_H_14_O	/	/	/	1.04
VOC56	17.972	Phenol, 2‐(1‐methylpropyl)‐	89–72‐5	C_10_H_14_O	/	0.17	/	/
VOC57	22.914	2,4‐Di‐tert‐butylphenol	96–76‐4	C_14_H_22_O	/	1.78	3.59	/
VOC58	22.956	Phenol, 2,4,6‐tris(1‐methylethyl)‐	2934‐07‐8	C_15_H_24_O	/	1.14	/	/
Total					9.45	7.11	4.86	6.01
Acids (10)								
VOC59	3.396	Acetic acid	64–19‐7	C_2_H_4_O_2_	/	/	/	0.27
VOC60	4.527	6‐Methoxy‐3‐methyl‐2‐benzofurancarboxylic acid	10410–29‐4	C_11_H_10_O_4_	/	0.05	/	/
VOC61	4.670	3,4‐Dimethoxycinnamic acid	2316–26‐9	C_11_H_12_O_4_	/	1.15	/	/
VOC62	4.815	4‐Diethylaminosalicylic acid	23050–90‐0	C_11_H_15_NO_3_	/	/	0.38	/
VOC63	9.009	8‐Phenylquinoline‐6‐carboxylic acid	35871–15‐9	C_16_H_11_NO_2_	/	/	0.81	/
VOC64	12.338	9,10‐Dihydrophenanthren‐2‐butyric acid	7494‐59‐9	C_18_H_18_O_2_	/	0.13	/	/
VOC65	14.881	2H‐Pyran‐2,6(3H)‐dione, dihydro‐4,4‐dimethyl‐	4160‐82‐1	C_7_H_10_O_3_	/	/	/	0.29
VOC66	19.451	Propanoic acid, 2‐methyl‐, 3‐hydroxy‐2,2,4‐trimethylpentyl ester	77–68‐9	C_12_H_24_O_3_	3.30	0.91	2.06	1.05
VOC67	22.907	[2,2’‐Bifuran]‐3‐carboxylic acid, 5′‐methyl‐, methyl ester	5896–31‐1	C_11_H_10_O_4_	/	/	/	0.30
VOC68	22.915	3,4‐Methylenedioxycinnamic acid	2373‐80‐0	C_10_H_8_O_4_	1.02	/	/	/
Total					4.32	3.24	3.25	1.91
Aldehydes (12)								
VOC69	10.241	Benzeneacetaldehyde	122–78‐1	C_8_H_8_O	/	/	/	0.59
VOC70	11.990	Nonanal	124–19‐6	C_9_H_18_O	12.60	2.52	7.89	4.71
VOC71	12.004	Furfural	98–01‐1	C_5_H_4_O_2_	/	0.26	/	/
VOC72	13.584	2‐Nonenal, (E)‐	18829–56‐6	C_9_H_16_O	4.19	/	3.18	/
VOC73	14.747	1,3‐Cyclohexadiene‐1‐carboxaldehyde, 2,6,6‐trimethyl‐	116–26‐7	C_10_H_14_O	/	/	/	0.43
VOC74	14.891	Decanal	112–31‐2	C_10_H_20_O	/	/	/	0.69
VOC75	15.279	Benzaldehyde, 4‐methyl‐	104–87‐0	C_8_H_8_O	/	0.32	/	/
VOC76	17.921	2,4‐Decadienal, (E,E)‐	25152–84‐5	C_10_H_16_O	/	/	1.97	4.43
VOC77	18.387	Piperonal	120–57‐0	C_8_H_6_O_3_	/	1.70	34.76	39.14
VOC78	19.167	2(3H)‐Furanone, 5‐heptyldihydro‐	104–67‐6	C_11_H_20_O_2_	/	/	/	0.31
VOC79	22.174	Benzaldehyde, 2,4‐dihydroxy‐3,6‐dimethyl‐	34883–14‐2	C_9_H1_0_O_3_	0.27	/	/	/
VOC80	22.189	Benzaldehyde, 3,4‐dimethoxy‐	120–14‐9	C_9_H_10_O_3_	/	55.50	1.15	/
Total					17.06	60.3	48.95	50.3
Ketones (18)								
VOC81	7.883	1,3,5‐Triphenyl‐1,5‐pentanedione	6263‐84‐9	C_23_H_20_O_2_	/	0.26	/	/
VOC82	9.506	1,2‐Propanedione, 1‐phenyl‐	579–07‐7	C_9_H_8_O_2_	/	/	0.42	/
VOC83	9.627	4,6‐Heptadiyn‐3‐one	29743–27‐9	C_7_H_6_O	/	/	/	0.42
VOC84	11.981	4‐Pyranone, 2,3‐dihydro‐	84302–42‐1	C_5_H_6_O_2_	2.08	/	/	/
VOC85	11.990	4(1H)‐Pyridone	108–96‐3	C_5_H_5_NO	1.77	/	/	/
VOC86	17.873	Ethanone, 1‐(2‐hydroxy‐5‐methylphenyl)‐	1450‐72‐2	C_9_H_10_O_2_	/	/	1.21	/
VOC87	18.297	5‐(p‐tert‐Butylphenoxymethyl)‐3‐(O‐tolyl)‐2‐oxazolidone	5198‐41‐4	C_21_H_25_NO_3_	0.15	/	/	/
VOC88	18.400	5,6,7,8‐Tetrahydro‐6‐oxopteridine	51036–16‐9	C_6_H_6_N_4_O	/	0.58	/	/
VOC89	20.350	2‐Hexen‐1‐one, 1‐(2‐hydroxy‐5‐methylphenyl)‐	51956–79‐7	C_13_H_16_O_2_	0.70	/	/	/
VOC90	21.453	5,9‐Undecadien‐2‐one, 6,10‐dimethyl‐, (E)‐	3796‐70‐1	C_13_H_22_O	2.38	0.60	1.69	1.21
VOC91	21.538	.delta.2‐1,3,4‐Oxadiazolin‐5‐one, 2‐(4‐pyridyl)‐	2845‐82‐1	C_7_H_5_N_3_O_2_	0.64	/	/	/
VOC92	22.167	3‐Butyn‐2‐one	1423‐60‐5	C_4_H_4_O	/	0.43	/	/
VOC93	22.600	7H‐Indeno[2,1‐a]anthracen‐7‐one	27582–45‐2	C_21_H_12_O	0.16	/	/	/
VOC94	24.242	Ethanone, 1,2‐diphenyl‐	451–40‐1	C_14_H_12_O	/	/	/	0.34
VOC95	24.936	Cyclopentanone, 2‐octyl‐	40566–23‐2	C_13_H_24_O	/	/	/	0.52
VOC96	26.560	3(2H)‐Benzofuranone, 6‐methoxy‐2‐[(3‐methoxyphenyl)methylene]‐, (E)‐	77764–84‐2	C_17_H_14_O_4_	/	0.28	/	/
VOC97	27.160	l‐Pyrrolid‐2‐one, N‐carbamoyl‐	40451–67‐0	C_5_H_8_N_2_O_2_	/	/	0.21	/
VOC98	29.635	4,4’‐Dihydroxybenzophenone	611–99‐4	C_13_H_10_O_3_	1.04	/	/	/
Total					8.92	2.15	3.53	2.49
Amines (14)								
VOC99	3.902	Methyl‐methoxy‐hydroxymethyl‐amine	6919‐52‐4	C_3_H_9_NO_2_	/	/	/	0.34
VOC100	6.709	Benzenamine, 4‐propyl‐	2696‐84‐6	C_9_H_13_N	/	0.05	/	/
VOC101	7.899	Benzenamine, 4‐(hexyloxy)‐	39905–57‐2	C_12_H_19_NO	/	/	/	0.40
VOC102	9.077	5‐Dimethylaminopyrimidine	31401–46‐4	C_6_H_9_N_3_	/	1.04	/	/
VOC103	11.979	L‐Prolinamide	7531‐52‐4	C_5_H_10_N_2_O	/	0.69	/	/
VOC104	11.985	Cyclopentanamine	1003‐03‐8	C_5_H_11_N	/	/	/	0.71
VOC105	14.710	Cyclohexanamine, N,N‐dimethyl‐	98–94‐2	C_8_H_17_N	/	0.36	/	/
VOC106	14.891	1,1‐Diethylpropargylamine	3234‐64‐8	C_7_H_13_N	0.48	/	/	/
VOC107	17.877	4‐Hydroxyphenylacetamide	17194–82‐0	C_8_H_9_NO_2_	/	0.48	/	/
VOC108	17.930	3‐(5‐Methylfuryl)‐N‐furamidopropionamide	331958–18‐0	C_13_H_14_N_2_O_4_	/	0.17	/	/
VOC109	24.512	Benzamide, N‐acetyl‐	1575‐95‐7	C_9_H_9_NO_2_	/	/	/	0.32
VOC110	24.923	1,3,5‐Triazine‐2,4,6‐triamine	108–78‐1	C_3_H_6_N_6_	/	/	0.38	/
VOC111	25.125	Benzamide, 4‐ethoxy‐	55836–71‐0	C_9_H_11_NO_2_	/	/	/	0.19
VOC112	29.645	Salicylanilide	87–17‐2	C_13_H_11_NO_2_	/	0.23	/	/
Total					0.48	3.02	0.38	1.96
Olefins (6)								
VOC113	13.584	2‐Octene, 4‐ethyl‐, (E)‐	74630–09‐4	C_10_H_20_	/	0.61	/	/
VOC114	17.972	5‐Pentylcyclohexa‐1,3‐diene	56318–84‐4	C_11_H_18_	/	/	1.35	/
VOC115	19.455	Cyclobuta[1,2‐d:3,4‐d’]bis[1,3]dioxole, tetrahydro‐, (3a.alpha.,3b.alpha.,6a.alpha.,6b.alpha.)‐	69956–59‐8	C_6_H_8_O_4_	/	0.25	/	/
VOC116	20.346	Longifolene	475–20‐7	C_15_H_24_	/	/	1.82	/
VOC117	22.193	Benzene, 3‐butenyl‐	768–56‐9	C_10_H_12_	/	0.32	/	/
VOC118	22.816	.alpha.‐Farnesene	502–61‐4	C_15_H_24_	/	/	/	0.94
Total					0	1.18	3.17	0.94
Heterocycles (30)								
VOC119	4.792	1,6‐Dimethylphenazine	58718–43‐7	C_14_H_12_N_2_	/	/	0.70	/
VOC120	6.663	2‐Ethyl‐3,5‐dimethylpyridine	1123‐96‐2	C_9_H_13_N	/	/	0.59	/
VOC121	6.682	2,5‐Dimethylpyrimidine	22868–76‐4	C_6_H_8_N_2_	/	0.54	/	/
VOC122	9.023	Quinoxaline, 2,3‐diphenyl‐	1684‐14‐6	C_20_H_14_N_2_	/	/	/	1.09
VOC123	9.508	5H‐1‐Pyrindine, 6,7‐dihydro‐	533–37‐9	C_8_H_9_N	/	0.34	/	/
VOC124	11.270	2‐Aminopurine	452–06‐2	C_5_H_5_N_5_	/	0.73	/	/
VOC125	11.984	Aziridine, 1,2,3‐trimethyl‐, trans‐	693–88‐9	C_5_H_11_N	/	/	/	1.26
VOC126	13.583	2‐(2‐Pyrrolidin‐1‐yl‐ethyl)‐pyridine	6311‐90‐6	C_11_H_16_N_2_	0.59	/	/	/
VOC127	14.250	Azulene	275–51‐4	C_10_H_8_	/	0.26	/	/
VOC128	14.656	Pyridine, 2‐pentyl‐	2294‐76‐0	C_10_H_15_N	/	/	/	0.62
VOC129	14.710	3‐Amino‐s‐triazole	61–82‐5	C_2_H_4_N_4_	/	/	0.68	/
VOC130	15.305	Benzofuran, 2,3‐dihydro‐	496–16‐2	C_8_H_8_O	/	/	/	0.92
VOC131	15.338	Benzofuran, 2‐ethenyl‐	7522‐79‐4	C_10_H_8_O	/	0.23	/	/
VOC132	15.667	7H‐Dibenzo[b,g]carbazole, 7‐methyl‐	3557‐49‐1	C_21_H_15_N	/	/	0.24	/
VOC133	16.238	6‐Methyl‐2‐phenyl‐7‐(2,4‐dimethylphenylmethyl)indolizine	64002–75‐1	C_24_H_23_N	0.31	/	/	/
VOC134	17.914	Furan, 2‐hexyl‐	3777‐70‐6	C_10_H_16_O	/	1.02	/	/
VOC135	17.958	2‐tert‐Butylpyridine	5944‐41‐2	C_9_H_1_3N	/	0.24	/	/
VOC136	18.278	1H‐Pyrazole, 4‐methyl‐3‐(4‐methylphenyl)‐1,5‐diphenyl‐	73306–07‐7	C_23_H_20_N_2_	/	0.22	/	/
VOC137	18.417	1,3‐Dioxolane, 2‐phenyl‐2‐(phenylmethyl)‐	4362‐19‐0	C_16_H_16_O_2_	/	/	/	13.82
VOC138	18.494	Benzofurazan	273–09‐6	C_6_H_4_N_2_O	/	/	/	0.15
VOC139	19.455	Cyclobuta[1,2‐d:3,4‐d’]bis[1,3]dioxole, tetrahydro‐, (3a.alpha.,3b.beta.,6a.beta.,6b.alpha.)‐	70004–63‐6	C_6_H_8_O_4_	/	0.24	/	/
VOC140	21.523	1H‐1,2,3‐Triazole, 4‐methyl‐5‐(5‐methyl‐1H‐pyrazol‐3‐yl)‐	51719–86‐9	C_7_H_9_N_5_	/	0.11	/	/
VOC141	22.656	1H‐Indole, 1,3‐dimethyl‐5,6‐dimethoxy‐2‐(3,5‐dimethoxyphenyl)‐	156785–76‐1	C_20_H_23_NO_4_	/	/	/	1.55
VOC142	22.906	3,4‐Dihydro‐4‐imino‐3‐methoxyquinazoline	15018–64‐1	C_9_H_9_N_3_O	/	/	/	0.24
VOC143	22.947	Furan, 2,5‐diphenyl‐	955–83‐9	C_16_H_12_O	/	/	0.32	/
VOC144	22.965	6‐Nitro‐5‐methoxy‐2,3‐dimethylindole	53918–83‐5	C_11_H_12_N_2_O_3_	/	0.19	/	/
VOC145	24.885	4‐Amino‐2‐(4′‐cyanobutyl)‐5,6‐trimethylene‐pyrimidine	30036–58‐9	C_12_H_16_N_4_	/	/	/	0.15
VOC146	24.940	2,2’‐Bifuran, octahydro‐	1592‐33‐2	C_8_H_14_O_2_	/	/	0.44	/
VOC147	26.550	1H‐Indole, 3‐(2‐methoxyethyl)‐2‐(2‐pyridyl)‐	161988–60‐9	C_16_H_16_N_2_O	0.15	/	/	/
VOC148	27.298	3‐Acetamidofuran	59445–85‐1	C_6_H_7_NO_2_	0.31	/	/	/
Total					1.36	4.12	2.97	19.8
Aromatic (17)								
VOC149	9.009	[1,1’‐Biphenyl]‐4‐carbonitrile, 4′‐ethyl‐	58743–75‐2	C_15_H_13_N	/	0.11	/	/
VOC150	9.624	Isopropyl phenyl ketone	611–70‐1	C_10_H_12_O	/	/	/	0.40
VOC151	12.334	Phenanthrene, 3,6‐dimethoxy‐9,10‐dimethyl‐	5025‐36‐5	C_18_H_18_O_2_	/	/	/	0.12
VOC152	13.510	[1,1’‐Biphenyl]‐4‐carbonitrile, 4′‐pentyl‐	40817–08‐1	C_18_H_19_N	0.22	/	/	/
VOC153	15.877	Benzene, pentamethyl‐	700–12‐9	C_11_H_16_	0.45	/	/	/
VOC154	16.780	1H‐Indene, 1‐ethyl‐2,3‐dihydro‐	4830‐99‐3	C_11_H_14_	/	0.17	/	/
VOC155	17.783	Naphthalene, 1‐methyl‐	90–12‐0	C_11_H_10_	0.43	/	0.77	/
VOC156	18.933	Naphthalene, 1,2‐dihydro‐1,1,6‐trimethyl‐	30364–38‐6	C_13_H_16_	/	0.30	/	/
VOC157	18.940	Naphthalene, 1,2‐dihydro‐2,5,7‐trimethyl‐	53156–03‐9	C_13_H_16_	/	/	/	0.18
VOC158	20.228	Naphthalene, 2,6‐dimethyl‐	581–42‐0	C_12_H_12_	/	/	0.28	/
VOC159	20.683	Naphthalene, 1,2‐dimethyl‐	573–98‐8	C_12_H_12_	/	/	0.17	/
VOC160	22.511	Fluorene	86–73‐7	C_13_H_10_	/	0.20	/	/
VOC161	22.961	Butylated hydroxytoluene	128–37‐0	C_15_H_24_O	/	/	3.32	/
VOC162	23.351	Naphthalene, 2,3,6‐trimethyl‐	829–26‐5	C_13_H_14_	/	0.19	/	/
VOC163	24.548	1‐Amino‐2‐methylnaphthalene	2246–44‐8	C_11_H_11_N	/	0.33	/	/
VOC164	26.810	1,1’‐Biphenyl, 2,2′,5,5′‐tetramethyl‐	3075‐84‐1	C_16_H_18_	2.27	0.44	/	/
VOC165	27.457	Anthracene, 1,2,3,4‐tetrahydro‐9,10‐dimethyl‐	94573–50‐9	C_16_H_18_	0.57	/	/	0.70
Total					3.94	1.74	4.54	1.40
Alcohols (4)								
VOC166	9.031	[3‐(4‐Methoxyphenyl)‐4,5‐dihydro‐1,2‐oxazol‐5‐yl]methanol	206055–84‐7	C_11_H_13_NO_3_	0.06	/	/	/
VOC167	17.869	1,2‐Benzisoxazol‐3(2H)‐one	21725–69‐9	C_7_H_5_NO_2_	/	0.93	/	/
VOC168	20.092	4‐Methyl‐1,6‐heptadien‐4‐ol	25201–40‐5	C_8_H_14_O	/	/	0.84	/
VOC169	25.127	Cedrol	77–53‐2	C_15_H_26_O	/	/	1.11	1.17
Total					0.06	0.93	1.95	1.17
Ethers (3)								
VOC170	6.570	3‐Dimethylaminoanisole	15799–79‐8	C_9_H_13_NO	/	0.11	/	/
VOC171	9.058	4‐Hexylanisole	81693–80‐3	C_13_H_20_O	/	0.05	/	/
VOC172	21.927	3‐tert‐Butyl‐4‐hydroxyanisole	121–00‐6	C_11_H_16_O_2_	/	/	/	0.53
Total					0	0.16	0	0.53
Oxides (2)								
VOC173	14.708	Di‐tert‐butyl peroxide	110–05‐4	C_8_H_18_O_2_	1.72	/	/	/
VOC174	26.806	1‐Methylphenazine 5‐oxide	14202–95‐0	C_13_H_10_N_2_O	0.47	/	/	/
Total					2.19	0	0	0
Quinones (1)								
VOC175	9.032	Anthraquinone, 2,3,6,7‐tetramethyl‐	15247–68‐4	C_18_H_16_O_2_	/	0.21	/	/
Others (6)								
VOC176	4.598	N‐Methylcoclaurine	3423‐07‐2	C_18_H_21_NO_3_	/	0.35	/	/
VOC177	4.673	Hydrastine	118–08‐1	C_21_H_21_NO_6_	/	/	0.26	/
VOC178	4.840	(.+/−.)‐Calycotomine	4356‐47‐2	C_12_H_17_NO_3_	/	0.21	0.48	/
VOC179	12.000	Acetaldehyde, butylhydrazone	20607–72‐1	C_6_H_14_N_2_	1.25	/	/	/
VOC180	13.566	2‐Cyanoguanidine	127099–85‐8	C_2_H_4_N_4_	/	/	0.83	/
VOC181	14.714	anti‐2‐Acetoxyacetaldoxime	37858–07‐4	C_4_H_7_NO_3_	/	/	0.88	/
Total					1.25	0.56	2.45	0

Abbreviations: AM, Raw Aohan millet; E‐AM, Expanded Aohan millet; F‐AM, Fried Aohan millet; S‐AM, Steamed Aohan millet.

### 3.6 Principal component analysis (PCA)

**FIGURE 2:**
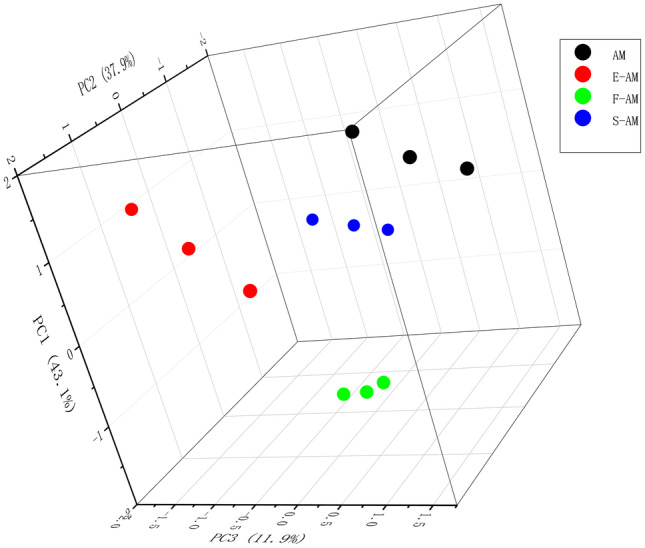
The PCA scatterplot of fatty acids in Aohan millet under different hot processing methods.

Principal component analysis (PCA) was performed to evaluate the impact of different thermal processing methods on Aohan millet. The PCA scores, plotted in three‐dimensions (3D), employed amino acid and fatty acid composition and content as classification criteria, as illustrated in Figure 2. The results revealed that the first principal component (PCA1) contributed 62.9% of the variance, second principal component (PCA2) contributed 19.6%, and third principal component (PCA3) contributed 10.3%. The cumulative contribution of the first three eigenvalues accounted for 92.8% of the total variance, indicating that the top three principal components encapsulated the bulk of the information from the indicators. Consequently, these top three eigenvalues were employed. The figure visually conveys that Aohan millet samples treated with different heating methods occupy distinct regions. Raw Aohan millet was positioned at the center, while the three distinct heat treatments induced significant divergence in amino acid and fatty acid composition and content in the millet. This implies that steamed, fried, and puffed Aohan millet exhibited dissimilarities in amino acid and fatty acid profiles, which offer opportunities for diverse Aohan millet product development.

### 3.7 Correlation analysis (amino acids and flavor substances, fatty acids, and flavor substances)

**FIGURE 3:**
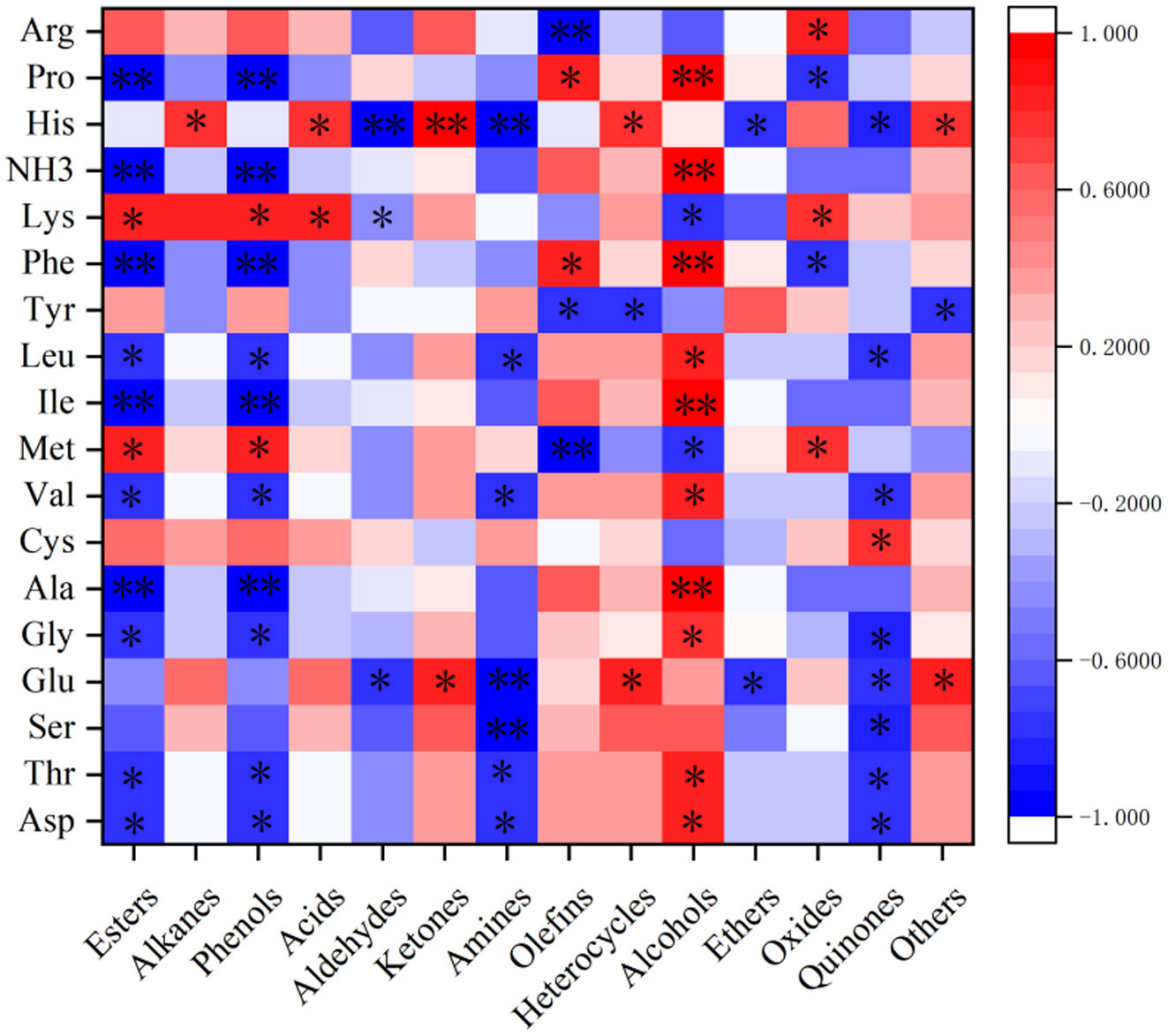
Correlation heat map between amino acids and flavor substances in Aohan millet under different hot processing methods. Red represents positive correlation, blue represents negative correlation, and *represents *p* < .05.

The results, as depicted in Figure 3, illustrated significant correlations between specific amino acids and volatile flavor substances. For instance, a highly significant positive correlation was identified between His and ketones (*p* < .01), whereas His exhibited a highly significant negative correlation with aldehydes and amines (*p* < .01). Additionally, Phe, Ile, and Ala displayed highly significant positive correlations with alcohols (*p* < .01) and highly significant negative correlations with esters and phenols (*p* < .01). Furthermore, several amino acids, including Ser, Glu, Cys, Tyr, His, and Arg, exhibited significant correlations with esters, phenols, and alcohols, as well as other amino acids (*p* < .05).

**FIGURE 4:**
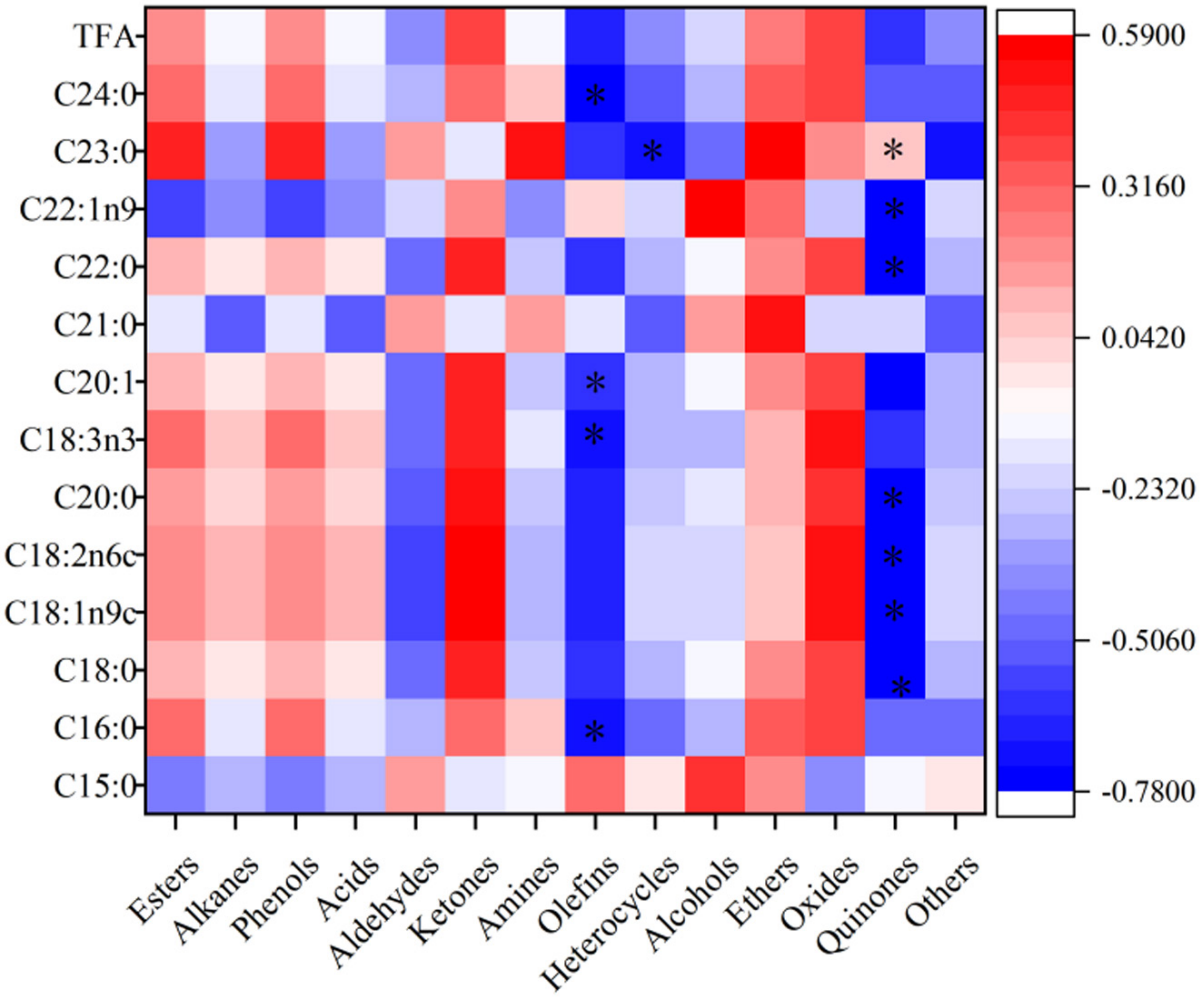
Correlation heat map between fatty acids and flavor substances in Aohan millet under different hot processing methods. Red represents positive correlation, blue represents negative correlation, and *represents *p* < .05.

The correlations between fatty acids and volatile flavor substances of Aohan millet were assessed using thermograms, depicted in Figure 4. Notably, C16:0, C18:3n3, C20:1, and C24:0 exhibited a significant negative correlation solely with olefins (*p* < .05). Meanwhile, C18:0, C18:1n9c, C18:2n6c, C20:0, C22:0, and C22:1n9 displayed a significant negative correlation with quinones, whereas C23:0 exhibited a significant positive correlation with quinones (*p* < .05). Furthermore, the relationship between C23:0 and heterocycles displayed a significant negative correlation (*p* < .05). The oxidation of fatty acids affects the aroma foxtail millet porridge. Therefore, unsaturated fatty acids are more conducive to the formation of Aohan millet flavor substances (Li, Zhao, Liu, Li, et al., 2021).

In summary, the study suggests that the production of volatile flavor compounds in Aohan millet was more closely related to amino acids than fatty acids.

## 4 CONCLUSION

Steamed millet exhibits elevated protein, fat, and starch content, thereby furnishing the body with essential nutrients and energy. Conversely, puffed millet shows reduced fat and starch levels, rendering it a suitable choice for individuals seeking weight loss. Furthermore, all three millet variants displayed diminished polyphenol and flavonoid concentrations relative to raw millet. Notably, fried and puffed millet exhibited increased amino acid and fatty acid content compared to steamed millet. After subjecting Aohan millet to steaming, there was a substantial increase in flavor compounds, rising from 53 to 80. In contrast, the other two groups did not demonstrate significant changes (*p* > .05).

The correlation analysis concluded that the connection between amino acids and flavor compounds in Aohan millet, under various thermal processing methods, was more robust than the relationship between fatty acids and flavor compounds. This suggests that the formation of flavor compounds was more closely linked to the types and quantities of amino acids.

Considering both fundamental nutrients and volatile flavor compounds, the steaming method proved to be the most effective. In contrast, the frying method was more effective when evaluating functional components, while the puffing method excelled in terms of amino acid and fatty acid content. The findings of this study offer current and valuable insights into influencing the flavor of Aohan millet during processing and selecting the most suitable processing approach. Nevertheless, the precise mechanistic underpinnings necessitate further exploration, involving an examination of millet's structural characteristics and potential enzyme alterations.

## AUTHOR CONTRIBUTIONS

**Likun Cheng:** Data curation (equal); funding acquisition (equal); methodology (equal); resources (lead); software (equal); writing – original draft (lead). **Shuang Qu:** Data curation (equal); formal analysis (equal); investigation (equal); visualization (equal). **Yueying Yun:** Data curation (equal); validation (lead). **Yan Ren:** Validation (equal). **Fucheng Guo:** Validation (equal). **Yakun Zhang:** Conceptualization (equal); supervision (equal); visualization (lead); writing – review and editing (lead). **Guoze Wang:** Conceptualization (equal); funding acquisition (lead); project administration (lead); supervision (equal).

## FUNDING INFORMATION

This work was financially supported by the 2022 Hondlon District Science and Technology Planning Project (YF2022016); The Fundamental Research Funds for Inner Mongolia University of Science & Technology (2023RCTD020, 2024QNJS019); The Special Research Project on Peak Carbon Dioxide Emissions and Carbon Neutralisation in Higher Education Institutions of Inner Mongolia (STZX202229).

## CONFLICT OF INTEREST STATEMENT

The authors declare that they have no known competing financial interests or personal relationships that could have appeared to influence the work reported in this paper.

## ETHICS STATEMENT

Ethics approval was not required for this research.

## Data Availability

The data presented in this study are available on request from the corresponding author.
